# Interventional Radiology in the Management of Primary Liver Malignancies

**DOI:** 10.3390/cancers18142283

**Published:** 2026-07-16

**Authors:** Kausthubh Hegde, Ronald Arellano, Shams Iqbal

**Affiliations:** Division of Interventional Radiology, Department of Radiology, Massachusetts General Hospital and Harvard Medical School, Boston, MA 02114, USA; rarellano@mgh.harvard.edu (R.A.); siiqbal@mgh.harvard.edu (S.I.)

**Keywords:** hepatocellular carcinoma, cholangiocarcinoma, interventional radiology, ablation, radioembolization, chemoembolization

## Abstract

Primary liver malignancies, including hepatocellular carcinoma, intrahepatic cholangiocarcinoma, and combined hepatocellular cholangiocarcinoma, remain major causes of cancer-related morbidity and mortality worldwide. Although systemic therapies have advanced substantially, intrahepatic tumor progression, liver failure, and portal hypertension continue to drive adverse outcomes in many patients. Interventional radiology plays a central and expanding role in the multidisciplinary management of these tumors by providing image-guided locoregional therapies for local tumor control, downstaging, bridging transplantation or resection, palliation, and potential survival benefit. This narrative review summarizes current evidence and technical considerations for major locoregional approaches, including radiofrequency ablation, microwave ablation, cryoablation, transarterial chemoembolization, transarterial radioembolization, endobiliary therapies, stereotactic body radiation therapy, irreversible electroporation, histotripsy, high-intensity focused ultrasound, hepatic arterial infusion, and brachytherapy. Interventional radiology also contributes to preoperative liver optimization through portal vein embolization, liver venous deprivation, lobar radioembolization to induce contralateral hypertrophy, and, in some patients, portal decompression before hepatic resection. For hepatocellular carcinoma, ablation and transarterial therapies are integrated into stage-based treatment algorithms and may provide curative-intent treatment in some patients. In intrahepatic cholangiocarcinoma, locoregional therapies provide meaningful disease control and may prolong survival, particularly when combined with systemic therapy. In combined hepatocellular cholangiocarcinoma, treatment remains individualized because of limited prospective data and heterogeneous tumor biology. Beyond cytoreduction, locoregional therapies can modulate the tumor immune microenvironment through immunogenic cell death, antigen release, cytokine signaling, and vascular remodeling, providing a rationale for combination strategies with immune checkpoint inhibitors, anti-angiogenic agents, and targeted therapies. As treatment paradigms evolve, the future of interventional radiology in primary liver cancer will depend on appropriate patient selection, optimized dosimetry and technique, integration with molecular and immunologic biomarkers, and coordinated multidisciplinary care.

## Simple Summary

Primary liver cancers, including hepatocellular carcinoma, intrahepatic cholangiocarcinoma, and combined hepatocellular cholangiocarcinoma, are often diagnosed when surgery or transplantation is not possible. Interventional radiology offers minimally invasive, image-guided treatments that can target tumors directly in the liver, control symptoms, keep bile ducts open, slow disease progression, and, in select patients, help make subsequent surgery or transplantation possible. Interventional radiology procedures can also enlarge the portion of the liver that will remain after surgery through portal vein embolization or liver venous deprivation and, in carefully selected patients, reduce elevated portal pressure before resection. This narrative review explains how ablation, transarterial chemoembolization, transarterial radioembolization, endobiliary treatments, radiation-based approaches, and newer technologies, such as irreversible electroporation and histotripsy, are used in the management of these cancers. It also discusses how local treatments can affect the immune environment of the tumor and potentially enhance the effects of immunotherapy and other systemic treatments. Careful patient selection, treatment technique, radiation-dose planning, and multidisciplinary coordination are essential to optimize outcomes.

## 1. Introduction

Primary malignancies of the liver are among the most lethal cancers worldwide, the majority of which are hepatocellular carcinoma (HCC) and intrahepatic cholangiocarcinoma (iCCA) [1]. According to GLOBOCAN 2022, primary liver cancers were the sixth most commonly diagnosed cancers and the third leading cause of cancer-related death worldwide, with an estimated 866,136 new cases and 758,725 deaths in 2022 [2]. HCC accounts for nearly 80% of cases, while iCCA represents 10–15%, and the rare combined hepatocellular cholangiocarcinoma (cHCC-CCA) accounts for less than 5% [3,4,5]. Despite their shared hepatic origin, these tumors differ in epidemiology, pathogenesis, morphology, stromal architecture, vascular supply, molecular profile, and immunologic landscape. These differences directly influence systemic and locoregional strategies for diagnosis, local control, downstaging, and palliation.

**Figure 1:**
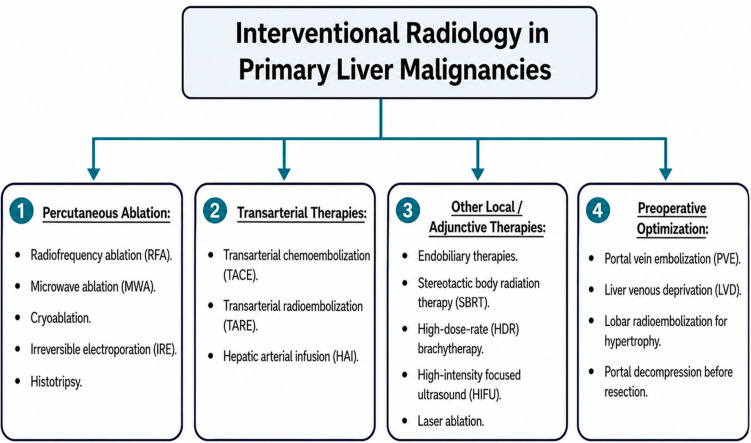
Major interventional radiology strategies for primary liver malignancies. Shown are the principal locoregional treatment categories and preoperative optimization approaches that may facilitate hepatic resection in selected patients.

Despite surveillance in at-risk populations, many patients with primary liver malignancies are diagnosed at intermediate or advanced stages, when resection or transplantation is not feasible [1,3]. Systemic therapies have advanced substantially, with tyrosine kinase inhibitors (TKIs), vascular endothelial growth factor (VEGF) inhibitors, programmed cell-death ligand 1 (PD-L1) inhibitors, immune checkpoint inhibitors (ICIs), and other targeted agents now standard in selected settings [6,7,8,9]. However, intrahepatic disease (through tumor progression, liver failure, biliary obstruction, or portal hypertension) remains the major cause of morbidity and mortality. Locoregional therapies (LRTs) [Figure 1], pioneered by interventional radiology (IR), offer a bridge between curative surgical strategies and systemic therapy by providing durable local tumor control, downstaging or bridging to transplantation or resection, palliation, and potential survival benefit. In addition, IR contributes to surgical candidacy through preoperative liver optimization strategies, including portal vein embolization, liver venous deprivation, and, in selected patients, portal decompression before hepatic resection.

Importantly, IR therapies and other LRTs are not merely cytoreductive. Ablation, embolization, and radiation also alter the tumor-immune axis. LRTs are capable of reshaping the hepatic tumor microenvironment by inducing immunogenic cell death (ICD), a process in which necrotic tumor cells release tumor-associated antigens (TAAs) and damage-associated molecular patterns (DAMPs) such as DNA, RNA, heat shock proteins (HSPs), adenosine triphosphate (ATP), high-mobility group box 1 (HMGB1), calreticulin, and uric acid [10]. These signals promote the recruitment and activation of dendritic cells (DCs), which then migrate to regional lymph nodes and present antigens with appropriate costimulatory signals to prime T lymphocytes. This cascade of events establishes both local and systemic antitumor immune responses, including the abscopal effect (a phenomenon by which LRTs not only shrink the targeted tumor but also lead to the shrinkage of untreated tumors elsewhere in the body) [11].

Conversely, sublethal or incomplete LRTs may generate a different response, characterized by inflammation, interleukin-6 (IL-6) and interleukin-8 (IL-8) release, and the upregulation of pro-oncogenic growth factors such as hypoxia-inducible factor-1α (HIF-1α), vascular endothelial growth factor (VEGF), hepatocyte growth factor (HGF), hepatocyte growth factor receptor (HGFR), and matrix metalloproteinases (MMPs), which together may facilitate angiogenesis, proliferation, and tumor progression [10]. The balance between these effects depends on technique, dose, extent of necrosis, and timing relative to systemic therapies. These effects provide a rationale for combining LRTs with immunotherapy, although direct evidence is strongest in HCC and remains less established in iCCA and cHCC–CCA [3,10].

This narrative review summarizes the role of locoregional and image-guided therapies in HCC, iCCA, and cHCC-CCA, with emphasis on technical considerations, patient selection, outcomes, safety, integration with systemic therapy, and the role of IR in preoperative liver optimization. Relevant English-language publications published through June 2026 were identified through searches of PubMed/MEDLINE and Google Scholar using combinations of terms related to ‘hepatocellular carcinoma’, ‘intrahepatic cholangiocarcinoma’, ‘combined hepatocellular-cholangiocarcinoma’, ‘interventional radiology’, ‘ablation’, ‘chemoembolization’, ‘radioembolization’, ‘endobiliary therapy’, ‘portal vein embolization’, ‘liver venous deprivation’, ‘radiation lobectomy’, and ‘immunotherapy’. Priority was given to professional-society guidelines, systematic reviews and meta-analyses, randomized and prospective studies, large retrospective cohort studies, and primary mechanistic studies. Additional publications were identified from the reference lists of relevant articles. Because this was a narrative synthesis, study selection was not conducted using a formal systematic-review protocol, and no quantitative meta-analysis or formal risk-of-bias assessment was performed.

This narrative review did not involve prospective recruitment, intervention, or systematic collection and analysis of patient-level data; therefore, institutional review board (IRB) approval was not required. All imaging examples are from the authors’ own clinical cases and were fully de-identified in accordance with the Health Insurance Portability and Accountability Act (HIPAA) requirements.

## 2. Hepatocellular Carcinoma (HCC)

**Figure 2:**
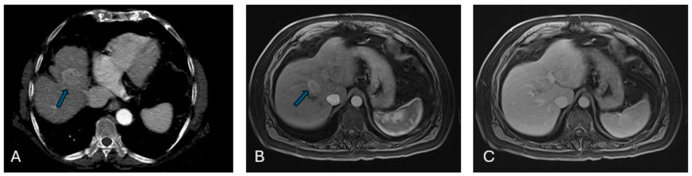
Axial contrast-enhanced computed tomography (CT) and magnetic resonance imaging (MRI) images demonstrating representative hepatocellular carcinoma (HCC) appearances. (**A**) CT scan from one patient demonstrates heterogeneous enhancement (arrow) in the right hepatic lobe, consistent with HCC. (**B**) MRI of a different patient demonstrates characteristic arterial hyperenhancement (arrow) of HCC with heterogeneous signal. (**C**) MRI from the same patient as in panel B demonstrates the characteristic washout of the HCC lesion.

HCC most often arises in cirrhotic livers due to chronic hepatitis B (particularly in East Asia and Africa), chronic hepatitis C (particularly in Western countries and Japan), alcohol-associated liver disease, or metabolic dysfunction-associated steatohepatitis [12]. Its pathogenesis involves cycles of necroinflammation, fibrogenesis, angiogenesis, and hepatocellular regeneration, leading to nodular transformation and malignant progression. On dynamic imaging, HCC typically demonstrates arterial-phase hyperenhancement with venous washout [12] [Figure 2].

Multiple professional societies have issued guidelines for the diagnosis and treatment of HCC [12,13,14,15]. While there are slight differences among them, all converge on the principle that optimal management requires a multidisciplinary approach. Management decisions are ideally undertaken in tumor boards that include diagnostic radiologists, hepatologists, interventional radiologists, medical oncologists, hepatobiliary and transplant surgeons, pathologists, and radiation oncologists.

Among the various staging systems proposed for HCC, the Barcelona Clinic Liver Cancer (BCLC) classification [16] is the most widely adopted framework because it couples prognostic stratification with evidence-based treatment allocation. The BCLC algorithm stratifies patients into five principal stages: very early (0), early (A), intermediate (B), advanced (C), and terminal (D), based on tumor burden, liver function, and Eastern Cooperative Oncology Group (ECOG) performance status [16,17]. Each stage is associated with a preferred first-line treatment strategy, ranging from curative-intent therapies such as surgical resection, ablation, and transplantation to transarterial modalities such as transarterial chemoembolization (TACE) and systemic therapy (Atezolizumab-Bevacizumab/Durvalumab-Tremelimumab). The BCLC system also emphasizes that HCC management is both algorithm-driven and individualized, requiring integration of clinical, biological, and patient-centered factors.

Ablative techniques such as radiofrequency ablation (RFA) and microwave ablation (MWA) are most effective in patients with very early or early-stage HCC, particularly when tumors are ≤3 cm, liver function is preserved, and resection or transplantation is not feasible [16]. Ablation may also be used as bridging therapy for transplant candidates because of organ-allocation constraints. Tumors near vulnerable structures may require adjunctive techniques such as hydrodissection, artificial ascites, artificial pleural effusion, or balloon protection before thermal ablation [18]. Transarterial chemoembolization (TACE) is typically used for intermediate-stage HCC for patients with multifocal liver-limited disease, preserved performance status, and adequate hepatic reserve, without vascular invasion or extrahepatic spread [16]. Transarterial radioembolization (TARE) is often utilized in patients with intermediate or advanced HCC who are less suitable for chemoembolization, particularly when portal vein thrombosis is present, and was added to the BCLC classification in the 2022 update. It is also being studied and used for downstaging or bridging to curative options such as resection or transplantation in select patients.

The major LRTs used in HCC are discussed below. Because reported outcomes across studies reflect differences in tumor burden, liver function, performance status, underlying liver-disease etiology, treatment era, patient selection, and management of recurrence, percentages reported for individual modalities should be interpreted within the context of each study and should not be directly compared across independent cohorts.

### 2.1. Radiofrequency Ablation (RFA)

RFA is one of the most extensively studied IR modalities for the treatment of small HCCs. It works by delivering high-frequency alternating current through an electrode to generate ionic agitation and frictional heating, raising tissue temperatures above 60 °C and inducing coagulative necrosis. The electrical circuit is completed through one or more grounding pads attached to the thighs or back of the patient [19].

RFA is a curative-intent option for appropriately selected patients with BCLC 0 or A disease when resection or transplantation is not feasible or appropriate [16]. Ideal candidates have preserved hepatic reserve, most commonly Child-Pugh class A or well-compensated Child-Pugh class B liver function, ECOG performance status 0–1, limited tumor burden, and no macrovascular invasion or extrahepatic disease [13,14,16]. RFA is best suited for solitary tumors ≤3 cm or up to three tumors each ≤3 cm, with the most favorable outcomes observed for lesions that are clearly visible on ultrasound, computed tomography (CT), magnetic resonance imaging (MRI), or fusion imaging. Tumors should be accessible by a safe percutaneous, laparoscopic, or intraoperative trajectory, and the anticipated ablation zone should encompass the lesion with an adequate circumferential margin while avoiding injury to central bile ducts, bowel, gallbladder, diaphragm, or major vascular structures.

Several technical factors influence patient selection and expected efficacy. RFA is less effective for tumors >3 cm, irregularly shaped lesions, and tumors adjacent to large vessels because of the heat-sink effect, in which flowing blood dissipates heat and may reduce the temperature achieved at the tumor margin. Lesions near the hepatic hilum or central bile ducts require caution because thermal injury can cause biliary stricture, biloma, or cholangitis. Tumors adjacent to the bowel, stomach, gallbladder, or diaphragm may still be treatable when protective techniques such as hydrodissection, artificial ascites, artificial pleural effusion, or balloon interposition can separate vulnerable structures from the planned ablation zone.

RFA may be performed percutaneously, laparoscopically, or intraoperatively with ultrasound, CT, or fusion imaging guidance [20,21,22]. Technical success depends on complete tumor coverage with an intended ablative margin of at least 5 mm, and preferably 5–10 mm when anatomically feasible, because microscopic satellite nodules are common around HCC [21,22]. Fusion imaging with CT or MRI may facilitate targeting of small or inconspicuous nodules. With conventional internally cooled RFA systems, energy is commonly delivered for approximately 6–12 min per application using an impedance-controlled or power-escalation algorithm; however, power settings, application duration, and treatment endpoints vary by generator, electrode design, and manufacturer protocol. For tumors larger than the expected ablation zone, the electrode may be repositioned, or multiple electrodes may be used, to create overlapping ablation zones that encompass the entire tumor and the intended ablative margin. The number and geometry of applications are determined by tumor size, shape, location, and the manufacturer-predicted ablation volume rather than by a fixed protocol.

Clinical outcomes after RFA are best for small, favorably located tumors. Multiple studies have demonstrated that RFA achieves high rates of complete tumor necrosis in carefully selected patients, with success rates exceeding 90% for lesions ≤3 cm and translating into favorable long-term outcomes. A 10-year survival analysis of more than 500 patients with solitary tumors ≤5 cm reported an overall survival of approximately 93% at 1 year, 63% at 5 years, and 45% at 10 years, with no significant difference between the treatment of initial and recurrent HCC [21]. While outcomes are best in tumors ≤3 cm, recurrence rates increase substantially for lesions >3 cm, with local tumor progression observed in 10–15% of cases within two to three years [21]. Comparative analyses suggest that RFA provides survival outcomes similar to surgical resection in early-stage HCC, although recurrence rates are generally higher, reflecting the limitations in margin control and the challenge of microscopic satellite disease [23].

RFA has a favorable safety profile and is less invasive than hepatic resection. Major complications occur in approximately 1–9% of cases and include hemorrhage, hepatic abscess, bile duct injury, diaphragmatic injury, and, rarely, tumor seeding, while procedure-related mortality remains below 0.5% [21]. Compared with hepatic resection, RFA offers shorter procedure time, reduced blood loss, fewer perioperative complications, and shorter hospitalization, supporting its role as a curative-intent therapy for very early and early HCC, particularly in patients who are not candidates for surgery.

### 2.2. Microwave Ablation (MWA)

MWA employs electromagnetic waves to agitate water molecules within tissue, creating frictional heat that damages cells via hyperthermic injury, leading to cell death by coagulative necrosis. Compared with RFA, MWA achieves higher temperatures, larger and more uniform ablation zones, and reduced susceptibility to the heat-sink effect, especially in areas near large blood vessels [24]. These properties make MWA particularly useful for tumors near large vessels and for selected tumors measuring 3–5 cm.

Patient selection for MWA overlaps substantially with RFA but includes a broader range of technically challenging tumors. MWA is most commonly used for BCLC 0 or selected BCLC A disease in patients with preserved performance status, adequate hepatic reserve, and no macrovascular invasion or extrahepatic spread [13,14,16]. Ideal candidates generally have ECOG performance status 0–1 and Child–Pugh class A liver function or carefully selected class B disease without clinically significant decompensation. It is particularly useful for tumors ≤3 cm and may also be considered for selected tumors measuring 3–5 cm when complete coverage and an adequate margin can be achieved using multiple antennas or overlapping applications. As with RFA, tumor visibility, safe access, coagulation status, proximity to adjacent structures, and transplant or resection candidacy should be assessed in a multidisciplinary setting.

MWA may be preferred over RFA for lesions adjacent to medium or large vessels because it is less affected by heat-sink-related cooling. It may also be advantageous for larger tumors requiring broader ablation zones, tumors with irregular geometry requiring multiple applicators, and for patients in whom shorter ablation time is desirable. However, the ability to generate larger and faster ablation zones also increases the importance of careful planning, particularly for tumors close to central bile ducts, bowel, gallbladder, diaphragm, or the hepatic capsule. Protective techniques such as hydrodissection, artificial ascites, artificial pleural effusion, and thermal monitoring may be required for tumors near vulnerable structures.

**Figure 3:**
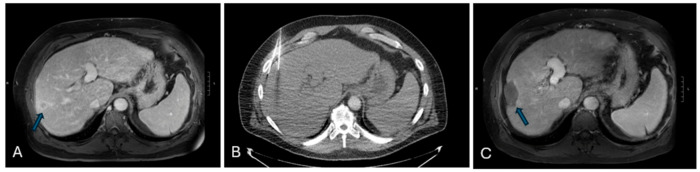
Axial imaging of hepatocellular carcinoma (HCC) treated with microwave ablation (MWA). (**A**) Pre-procedural contrast-enhanced magnetic resonance imaging (MRI) demonstrates an arterially enhancing lesion with washout (arrow) consistent with HCC in the right hepatic lobe. (**B**) Intra-procedural computed tomography (CT) showing placement of the microwave ablation probes into the lesion. (**C**) Post-ablation contrast-enhanced MRI demonstrates a non-enhancing ablation zone (arrow) encompassing the target tumor, consistent with complete treatment.

The procedure involves image-guided placement of one or more microwave antennas within the tumor [Figure 3]. Ultrasound, CT, cone-beam CT, MRI fusion, or multimodality guidance may be used depending on tumor conspicuity and institutional practice. Power and application time are selected according to the microwave platform, antenna design, tumor dimensions, tissue characteristics, and the required ablative margin. Reported clinical protocols vary substantially by device and generally use approximately 60–150 W for about 3–10 min per antenna application, although these settings are not interchangeable among platforms. Contemporary high-power systems commonly operate at 100–150 W and may produce the intended ablation volume within approximately 3–5 min; for example, a recent HCC study reported mean application times of approximately 3.8 min at 100 W and 3.0 min at 150 W [25]. Multiple antennas may be activated simultaneously or sequentially to enlarge the ablation zone and conform treatment to irregular tumor geometry. Final parameters should follow manufacturer-specific ablation charts and be adjusted using intraprocedural imaging to ensure coverage of the tumor and intended margin while protecting adjacent structures.

Clinical outcomes after MWA are favorable, particularly for tumors ≤3 cm. A meta-analysis of nine studies involving 2381 patients compared ultrasound-guided MWA for HCC at specific anatomical sites with MWA for HCC at non-specific sites. Complete ablation, major complication rates, and the 1-, 3-, and 5-year overall survival did not differ significantly between groups. Among the four studies reporting 5-year survival, the pooled rates were 42.5% for tumors at specific anatomical sites and 40.0% for tumors at non-specific sites [26]. These values reflect the specific cohorts included in that analysis and should be interpreted within that context rather than as universal survival estimates for MWA. Overall, the available evidence supports MWA as an effective treatment for early HCC and as a particularly useful option for lesions in locations where larger ablation zones or reduced heat-sink susceptibility are advantageous.

Safety outcomes following MWA are generally favorable, with major complications reported in approximately 2–4% of patients [26]. These events are predominantly focal procedural complications and include hemorrhage, biliary injury or stricture, hepatic abscess or other infection, pleural complications, gastrointestinal injury, skin injury, and, rarely, tumor seeding. Although uncommon, some of these events may require drainage, embolization, surgery, or prolonged hospitalization. Risk is influenced by tumor size and location, the number and configuration of antenna applications, and proximity to central bile ducts or adjacent organs. Compared with RFA, MWA offers faster heating, larger ablation zones, and less heat-sink effect; however, careful treatment planning is essential because larger ablation zones may increase the risk of collateral injury when tumors are close to critical structures.

### 2.3. Cryoablation

Cryoablation employs rapid cooling, typically using liquid nitrogen or argon-based systems, to achieve cytotoxic tissue temperatures below −40 °C, resulting in intracellular ice formation, osmotic stress, and vascular thrombosis. Unlike heat-based ablation, cryoablation creates a visible ice ball on CT, ultrasound, or MRI, allowing real-time monitoring of the ablation zone and its relationship to adjacent structures [27]. This visual feedback is a major technical advantage.

Patient selection criteria overlap with those for RFA and MWA, but cryoablation is particularly useful in selected patients with tumors in high-risk locations. Appropriate candidates generally have early-stage or limited HCC, preserved functional status, adequate hepatic reserve, and no extrahepatic disease or macrovascular invasion when curative-intent treatment is planned. Patients should typically have Child–Pugh class A or carefully selected class B liver function, acceptable coagulation parameters, and sufficient residual hepatic reserve. Cryoablation may be considered when heat-based ablation is less suitable because of lesion location, anticipated pain, or need for precise visualization of the ablation zone [28]. However, caution is required for tumors near central bile ducts, bowel, gallbladder, or major vascular structures, and protective displacement techniques may still be needed. Cryoablation may be less desirable in patients with severely limited hepatic reserve, coagulopathy, thrombocytopenia, or large tumors requiring extensive freeze volumes because larger ablation volumes may increase the risk of bleeding, hepatic decompensation, and systemic inflammatory complications.

The procedure is performed by placing one or more cryoprobes into the tumor under image guidance. Treatment typically consists of freeze–thaw cycles, with probe number and configuration determined by tumor size, shape, and desired margin. The visible ice ball should extend beyond the tumor margin to ensure adequate cytotoxic coverage, although the lethal isotherm lies within the visible ice-ball edge; therefore, the visible margin must be planned accordingly. Cryoablation can also be performed percutaneously or surgically.

Clinical data support cryoablation as an effective option in selected patients, including those with lesions near critical structures. In a large cohort of 324 patients, including 106 with high-risk lesions adjacent to critical structures, CT-guided percutaneous cryoablation achieved a 100% technical success rate with 12- and 24-month complete ablation rates of 82–84% and 72–74%, respectively [28]. In a large single-center cohort of patients with HCC within Milan criteria, percutaneous cryoablation was associated with favorable long-term outcomes and a 5-year overall survival rate of approximately 60% [29].

The safety profile of modern image-guided hepatic cryoablation is generally favorable, with pooled major complication rates around 4–5% [30]. Complications include hemorrhage, biliary injury, hepatic abscess, pleural complications, hepatic decompensation, and injury to adjacent structures. Cryoablation also carries a distinctive risk of cryoshock, a rare but potentially life-threatening systemic inflammatory response characterized by hemodynamic instability, coagulopathy, and possible renal, respiratory, or multiorgan dysfunction. A recent meta-analysis estimated the incidence of cryoshock at approximately 0.27%, with no reported cases after treatment of lesions smaller than 3 cm [30].

Comparative data suggest that cryoablation provides local tumor control and survival outcomes broadly comparable to RFA in selected patients. In a multicenter randomized trial of patients with one or two HCCs measuring ≤4 cm, 5-year overall survival was similar after cryoablation and RFA, at 40% vs. 38%, respectively, without a demonstrated overall survival advantage. Cryoablation was associated with lower local tumor progression, particularly for tumors >3 cm [31]. Direct comparative evidence between cryoablation and MWA remains limited. Overall, cryoablation is best viewed as a complementary ablation modality rather than a replacement for RFA or MWA, with particular value for selected tumors in high-risk locations and cases in which real-time ice-ball visualization is advantageous.

### 2.4. Transarterial Chemoembolization (TACE)

TACE is a standard locoregional therapy for appropriately selected patients with intermediate-stage HCC, particularly BCLC B disease, who are not candidates for ablation, curative resection, or transplantation [13,14,16,32]. TACE exploits the preferential arterial supply of HCC relative to background liver parenchyma by delivering intra-arterial chemotherapy followed by arterial embolization, thereby combining cytotoxic drug exposure with ischemic tumor injury. Conventional TACE (cTACE) typically uses an emulsion of ethiodized oil and chemotherapy (doxorubicin, cisplatin, or mitomycin), followed by embolic agents such as gelatin sponge or polyvinyl alcohol particles. Drug-eluting bead TACE (DEB-TACE) uses calibrated microspheres that release chemotherapy in a controlled manner, reducing systemic toxicity [32,33]. Superselective catheterization using microcatheters is critical to maximize tumor necrosis while preserving surrounding parenchyma.

Patient selection is central to the safety and efficacy of TACE. Appropriate candidates generally have intermediate-stage, liver-limited HCC; ECOG performance status 0; preserved hepatic reserve, most commonly Child-Pugh class A or carefully selected Child–Pugh class B liver function; and no clinically significant decompensation [13,14,16]. TACE is best suited for patients with multinodular disease that is not amenable to curative-intent ablation or resection but in whom tumor burden remains compatible with preserved hepatic function. Patients with portal hypertension may still be candidates if liver function is compensated, but decompensated cirrhosis, refractory ascites, uncontrolled encephalopathy, severe jaundice, poor performance status, extensive bilobar tumor burden, and extrahepatic spread generally favor systemic therapy or supportive care rather than TACE [16,32,34].

Vascular invasion requires individualized assessment. Main portal vein thrombosis has historically been considered a contraindication to TACE because embolization can precipitate hepatic ischemia or liver failure, particularly in patients with impaired portal perfusion or limited hepatic reserve. However, selected patients with segmental or subsegmental portal vein tumor thrombus, preserved liver function, and adequate collateral portal flow may still be considered for carefully tailored treatment in experienced centers. In general, transarterial radioembolization (TARE) or systemic therapy is preferred when clinically significant portal vein invasion is present [16,32]. Other important contraindications or relative contraindications include bilirubin >2 mg/dL, severe arterioportal shunting, untreated biliary obstruction, active infection, renal insufficiency that precludes angiography, uncorrectable coagulopathy, and inability to catheterize the tumor-feeding artery safely [16,32,33].

The procedure involves selective catheterization of the hepatic arterial branches supplying the tumor, usually with a microcatheter. Superselective or subsegmental delivery is preferred whenever feasible because it maximizes intratumoral drug delivery and ischemia while minimizing injury to non-tumoral liver parenchyma. Cone-beam CT can improve tumor-feeder identification, confirm catheter position, and assess treatment coverage during the procedure [32,33]. The endpoint varies according to technique and institutional practice but typically involves delivery until near-stasis or substantial reduction in tumor arterial flow while avoiding reflux into non-target branches. Repeat TACE sessions are frequently required for multifocal disease, but retreatment should be based on radiologic response, residual viable tumor, liver function, and clinical tolerance rather than a fixed schedule. Patients who develop progressive hepatic dysfunction, worsening performance status, or TACE-refractory disease should be transitioned to systemic therapy rather than undergoing repeated nonbeneficial embolization [35].

Randomized trials and meta-analyses established that TACE improves survival compared with best supportive care in appropriately selected patients with unresectable HCC, supporting its role as a locoregional therapy for intermediate-stage disease [36,37]. Contemporary series generally report median overall survival of approximately 20–30 months in selected BCLC B cohorts, although outcomes vary substantially according to tumor burden, Child-Pugh class, albumin-bilirubin grade, portal hypertension, treatment selectivity, and response to therapy [38,39]. Complete pathologic necrosis is uncommon after conventional lobar or segmental TACE, but objective responses and durable disease control can be achieved, particularly with superselective treatment of limited tumor burden [32,33].

TACE is generally safe when performed in carefully selected patients, but toxicity is closely linked to baseline hepatic reserve and treatment extent. Postembolization syndrome, characterized by fever, abdominal pain, nausea, and transient transaminase elevation, is common and usually self-limited. Major complications occur in a minority of patients and include hepatic failure, hepatic abscess, biliary injury, gallbladder ischemia, gastrointestinal ulceration from non-target embolization, vascular injury, renal dysfunction, and infection [32,33]. Procedure-related mortality is generally low in contemporary practice but increases substantially in patients with decompensated cirrhosis, high bilirubin, extensive tumor burden, or nonselective embolization. Compared with ablation, TACE is better suited for multifocal intermediate-stage disease but is less commonly curative. Compared with TARE, TACE has a stronger historical evidence base in BCLC B disease but is associated with more postembolization symptoms and may be less suitable in patients with portal vein invasion or limited tolerance for ischemic injury.

### 2.5. Transarterial Radioembolization (TARE)

TARE, also referred to as selective internal radiation therapy (SIRT), is an intra-arterial therapy that delivers yttrium-90 (Y90)-loaded microspheres into the hepatic arterial branches supplying the HCC. Y-90 is a beta-emitting radioisotope that produces high-dose internal radiation with limited tissue penetration, allowing preferential irradiation of the tumor-bearing liver while relatively sparing uninvolved parenchyma. Unlike TACE, TARE produces minimal macroembolic ischemia and is therefore particularly useful in selected patients with portal vein tumor thrombus or those who may not tolerate ischemic embolization [40].

Two principal Y-90 microsphere platforms are commercially available and used clinically: glass microspheres (TheraSphere^®^) with high specific activity per sphere and resin microspheres (SIR-Spheres^®^) with lower specific activity but higher embolic load [40]. Treatment planning requires meticulous mapping angiography to define hepatic arterial anatomy, identify tumor-feeding vessels, and exclude extrahepatic branches that could lead to non-target radiation. Technetium-99 m macroaggregated albumin (99 mTc-MAA) scanning is used to estimate lung shunt fraction and assess the risk of extrahepatic deposition. Prophylactic embolization of selected extrahepatic branches may be performed when necessary, although contemporary practice increasingly relies on catheter positioning and cone-beam CT rather than routine embolization alone.

Patient selection for TARE includes patients with unresectable HCC, preserved performance status, adequate hepatic reserve, and liver-dominant disease. TARE may be used in selected patients with BCLC A disease who are not candidates for ablation, resection, or transplantation; in selected BCLC B patients as an alternative to TACE; and in selected BCLC C patients with portal vein tumor thrombus when liver function is preserved and extrahepatic disease is absent or limited [13,14,16,40]. Ideal candidates generally have ECOG performance status 0–1, Child-Pugh class A or carefully selected Child-Pugh class B liver function, acceptable bilirubin, and tumor distribution that can be treated while preserving adequate functional liver volume. Relative contraindications include poor performance status, decompensated cirrhosis, marked hyperbilirubinemia, high lung shunt fraction or estimated excessive lung dose, uncorrectable flow to the gastrointestinal tract, extensive tumor burden involving most of the liver, and inadequate residual hepatic reserve [40].

Dosimetry is a central determinant of efficacy and safety. Treatment may be planned using body surface area, medical internal radiation dose, partition, multicompartment, or voxel-based approaches, depending on microsphere type, institutional practice, and available imaging. Increasingly, personalized dosimetry is favored because tumor absorbed dose, non-tumoral liver dose, and treated-volume dose correlate with response and toxicity. For glass microspheres, tumor absorbed doses around or above 190–205 Gy have been associated with improved response and local tumor control, whereas radiation segmentectomy uses ablative dosing, commonly targeting a perfused-volume dose >400 Gy in a limited segmental or subsegmental territory [40,41,42]. Lung dose constraints, commonly ≤30 Gy in a single treatment and ≤50 Gy cumulatively, are used to reduce the risk of radiation pneumonitis [40].

**Figure 4:**
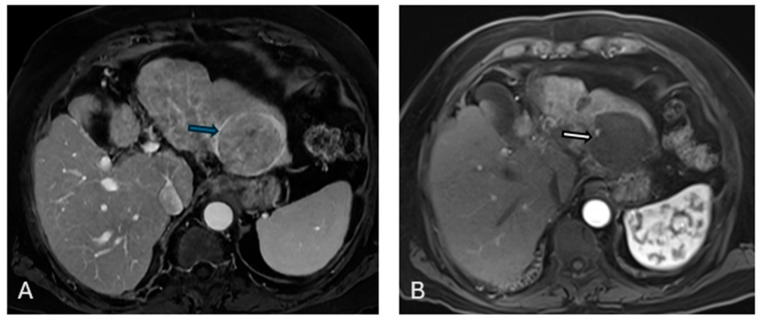
Axial contrast-enhanced magnetic resonance imaging (MRI) demonstrating treatment response after radiation segmentectomy. (**A**) Pre-treatment image shows a large arterially enhancing mass (arrow) consistent with hepatocellular carcinoma (HCC). (**B**) Post-treatment follow-up demonstrates necrosis and lack of enhancement within the treated segment (arrow), consistent with successful radiation segmentectomy.

The technique is tailored to tumor distribution. Lobar TARE may be used for multifocal lobar disease, whereas segmental or subsegmental delivery can be used for limited disease. Radiation segmentectomy, also termed ablative radioembolization, delivers a high radiation dose to one or two Couinaud segments with the goal of complete tumor necrosis while sparing the remaining liver [Figure 4]. This approach is particularly useful for solitary tumors that are not amenable to thermal ablation because of size, location, poor sonographic visibility, or proximity to vessels, bile ducts, or the diaphragm. Lobar radioembolization can also induce contralateral hypertrophy, a strategy sometimes referred to as radiation lobectomy and may contribute to future resection candidacy in selected patients.

Clinical evidence supports TARE as an effective locoregional therapy across selected HCC populations. In the PREMIERE trial, TARE significantly prolonged time to progression compared with cTACE while overall survival was similar [43]. The LEGACY study of Y-90 glass microspheres in solitary unresectable HCC reported an objective response rate of 88.3%, a durable response in 62.2%, and a 3-year overall survival of 86.6% in the overall cohort, with higher survival among patients who subsequently underwent resection or transplantation [44]. Radiation segmentectomy series have also demonstrated high rates of complete pathologic necrosis when ablative dose thresholds are achieved, particularly with absorbed doses >400 Gy [41,42].

TARE is generally well tolerated. Common side effects include fatigue, nausea, abdominal discomfort, anorexia, and transient liver enzyme elevation. Major complications are uncommon but include radioembolization-induced liver disease, hepatic decompensation, biliary injury, abscess in patients with altered biliary anatomy, radiation cholecystitis, gastrointestinal ulceration from non-target microsphere deposition, and radiation pneumonitis in the setting of excessive lung shunting. Compared with TACE, TARE is associated with fewer postembolization symptoms and longer time to progression in selected patients, but it requires more complex planning, careful dosimetry, and attention to radiation safety. TARE is therefore best viewed as complementary to TACE rather than interchangeable with it, with modality selection guided by tumor distribution, portal vein patency, liver function, treatment intent, dosimetry feasibility, and institutional expertise.

### 2.6. Other Modalities

Stereotactic body radiation therapy (SBRT) is a noninvasive treatment that delivers highly conformal, high-dose radiation to hepatic tumors over a limited number of fractions. It is most useful for patients with limited disease who are not candidates for ablation, resection, transplantation, or transarterial therapy because of tumor location, limited percutaneous access, vascular proximity, or comorbidity. In five-fraction SBRT for HCC, a planning target volume D95% ≥40 Gy has been associated with improved local control, with reported 2-year local control rates exceeding 80% in selected patients [45]. Toxicity is closely related to baseline hepatic reserve and irradiated liver volume; therefore, careful patient selection and strict dose-volume constraints are essential to reduce the risk of radiation-induced liver disease and hepatic decompensation.

Irreversible electroporation (IRE) is a nonthermal ablative technique that applies high-voltage electrical pulses across tumors to induce apoptosis while sparing bile ducts and vascular structures. This property makes IRE a potential option for centrally located tumors or tumors near major vascular or biliary structures, where thermal ablation may carry a higher risk. Technical execution requires parallel electrode placement and cardiac synchronization to reduce the risk of arrhythmias. Current evidence in HCC remains limited compared with RFA, MWA, TACE, and TARE, but early studies and reviews suggest that IRE can provide local control in selected technically challenging tumors [46,47].

Histotripsy is an emerging non-invasive, non-thermal focused ultrasound technique that mechanically disrupts liver tumors through acoustic cavitation. The Edison System (HistoSonics, Minneapolis, MN, USA) received FDA De Novo authorization in 2023 [48,49] for noninvasive destruction of liver tumors, including unresectable liver tumors, using a nonthermal mechanical process, but its role in HCC treatment algorithms remains under investigation. Histotripsy may preserve native tumor antigens and stimulate systemic antitumor immune responses, raising interest in future combinations with immune checkpoint inhibitors. However, clinical evidence for histotripsy in HCC remains early, and its use should currently be considered investigational or highly selected outside established clinical pathways [50].

High-intensity focused ultrasound (HIFU) and laser ablation remain largely investigational in HCC, with limited availability and variable reproducibility. Each, however, represents an avenue of future exploration, particularly as adjuncts in patients who are not candidates for more established locoregional approaches [46].

## 3. Intrahepatic Cholangiocarcinoma (iCCA)

**Figure 5:**
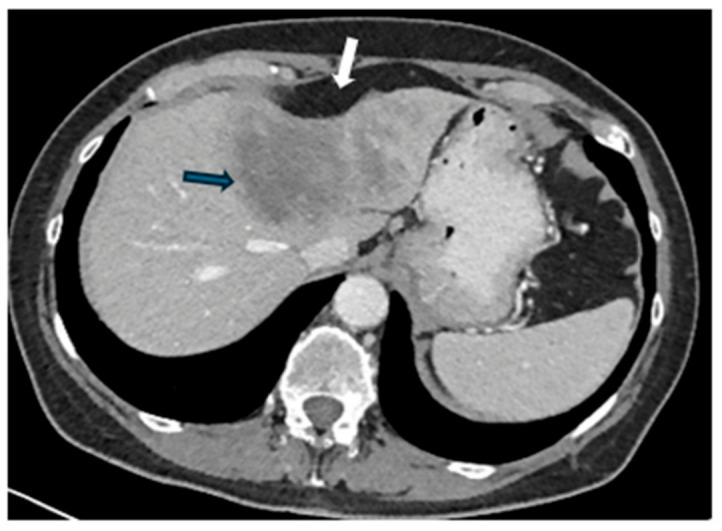
Axial contrast-enhanced computed tomography (CT) image demonstrating intrahepatic cholangiocarcinoma, seen as a large hypoenhancing mass (dark arrow) in the right hepatic lobe with irregular margins and associated capsular retraction (white arrow).

Cholangiocarcinomas (CCAs) are a heterogeneous group of malignancies that arise from the cholangiocytes that line the biliary tree. CCAs are classified based on their anatomic location, as follows: (1) intrahepatic CCA (iCCA), (2) perihilar CCA (pCCA), or (3) distal CCA (dCCA) [51]. Intrahepatic cholangiocarcinoma (iCCA) [Figure 5] is considered a primary liver malignancy and is the subtype most relevant to image-guided liver-directed therapies. It arises from biliary epithelial cells within a dense desmoplastic stroma.

Established risk factors include primary sclerosing cholangitis, hepatolithiasis, congenital biliary anomalies, chronic viral hepatitis, cirrhosis, and liver fluke infection in endemic regions [51]. On imaging, tumors often demonstrate peripheral rim enhancement with progressive central filling, reflecting their hypoperfused, fibrotic microenvironment. Prognosis remains poor because most patients present with advanced or unresectable disease. Fewer than one-third of patients are candidates for curative-intent resection at presentation, and recurrence after surgery is common, with reported 5-year overall survival generally ranging from approximately 20% to 45% [52,53].

Systemic therapy is central for unresectable or metastatic disease. The ABC-02 trial [54] established gemcitabine-cisplatin as the first-line standard, while ABC-06 [55] supported FOLFOX as a second line. More recently, TOPAZ-1 [56] demonstrated improved survival with the addition of durvalumab to gemcitabine and cisplatin. Targeted therapy is available in biomarker-selected subgroups, including pemigatinib for fibroblast growth factor receptor 2 (FGFR2) alterations and ivosidenib for isocitrate dehydrogenase 1 (IDH1) mutant disease. Zanidatamab has also been approved for advanced cholangiocarcinoma that is HER2-positive [57]. Despite these advances, intrahepatic progression remains a major cause of morbidity and mortality, highlighting the need for locoregional therapies as part of multimodal management.

### 3.1. Thermal Ablation

Thermal ablation has a limited but defined role in iCCA, primarily for small, solitary tumors or limited intrahepatic recurrence in patients who are not candidates for surgery. RFA and MWA are the most commonly reported modalities, with the best outcomes observed for tumors ≤3 cm, preserved hepatic function, and absence of extrahepatic disease [58,59]. Because iCCA is often infiltrative and may be associated with microscopic satellite disease, wider ablative margins, ideally approaching 1 cm, are desirable when anatomically feasible [59,60]. However, efficacy declines for larger tumors because of irregular margins, desmoplastic stroma, and difficulty achieving complete tumor coverage.

For RFA, reported technical success rates range from approximately 80% to 100%, with median overall survival ranging from approximately 20 to 52 months in highly selected patients [58,59,60]. Comparative data suggest that surgical resection generally provides superior survival when feasible; however, RFA may offer a reasonable alternative in selected patients with small tumors who are not surgical candidates [61].

MWA may offer practical advantages over RFA by creating larger and more homogeneous ablation zones that are less affected by vascular flow. Patients typically include those with tumors up to 5 cm, recurrent disease after resection, or multifocal lesions in otherwise preserved liver function. Reported median overall survival after MWA ranges from approximately 10 to 32 months in most recurrent and unresectable iCCA series [59,62].

Recent multicenter data in patients with one or two iCCA lesions ≤35 mm treated with monopolar RFA or MWA reported a median disease-free survival of 10.5 months and a median overall survival of 40.8 months [63]. Some comparative analyses suggest that ablation may provide outcomes comparable to repeat surgery for selected intrahepatic recurrences with fewer complications; however, these findings are limited by retrospective design and selection bias [62].

Cryoablation has been reported less frequently for iCCA but may be considered for selected anatomically challenging lesions, including tumors near vascular, capsular, or biliary structures where real-time visualization of the ice ball may be advantageous.

Major complications after ablation are uncommon but include hepatic abscess, bile leak, cholangitis, hemorrhage, and injury to adjacent structures, with higher infectious risk in patients with biliary-enteric anastomoses or indwelling biliary stents. Cryoablation may very rarely cause cryoshock, particularly following large-volume hepatic ablation, as discussed in the HCC section. In a meta-analysis of thermal ablation for iCCA, pooled 1-, 3-, and 5-year overall survival rates were 82.4%, 42.1%, and 28.5%, respectively, and the pooled major complication rate was 5.7% [58]. Overall, ablation should be considered a selective option for small-volume iCCA rather than a broadly applicable substitute for resection or systemic therapy.

### 3.2. Transarterial Chemoembolization (TACE)

TACE has been used for unresectable, liver-dominant iCCA in patients with preserved liver function and performance status. Compared with HCC, iCCA is often less hypervascular and more desmoplastic, which may limit the effectiveness of embolization-based therapy. Nevertheless, studies suggest that TACE can provide meaningful disease control in selected patients. A pooled analysis of 19 studies, including 1091 patients, reported disease-control rates of 72.8% with conventional TACE and 88.7% with DEB-TACE, while another meta-analysis reported a median overall survival of 14.2 months after TACE [64,65]. Reported median overall survival across individual series generally ranges from approximately 12 to 20 months, reflecting differences in tumor burden, prior systemic therapy, embolic technique, and patient selection [64,65].

DEB-TACE has shown higher objective response and disease-control rates than conventional TACE in some cohorts, but a consistent overall survival advantage has not been established. In a pooled analysis, objective response rates were 51.2% with DEB-TACE and 29.4% with conventional TACE, whereas the difference in overall survival was not significant [64]. However, comparative data remain heterogeneous, and no single TACE approach has been established as standard for iCCA.

Patient selection is particularly important. Appropriate candidates have liver-dominant disease, preserved liver function, limited tumor burden, and no uncontrolled biliary obstruction or active cholangitis. Patients with biliary instrumentation, bilioenteric anastomosis, or biliary obstruction are at increased risk for hepatic abscess and infectious complications. Combination strategies, including TACE with systemic therapy or targeted agents, have shown encouraging results in selected cohorts. In one study, apatinib plus DEB-TACE was associated with longer median overall survival than apatinib alone (19.3 vs. 6.5 months), but these data remain nonrandomized and should be interpreted cautiously [66].

### 3.3. Transarterial Radioembolization (TARE)

**Figure 6:**
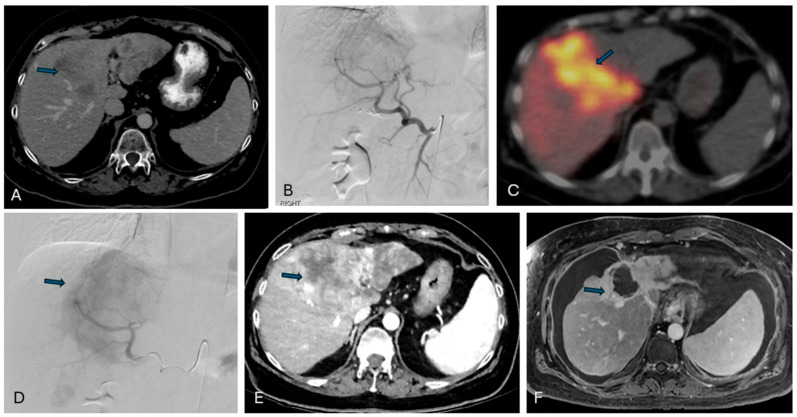
Sequential imaging and angiography demonstrating treatment of intrahepatic cholangiocarcinoma with Yttrium-90 (Y-90) radioembolization. (**A**) Baseline contrast-enhanced computed tomography (CT) shows a large right hepatic lobe cholangiocarcinoma (arrow). (**B**) Planning angiogram demonstrates arterial anatomy for selective catheterization. (**C**) Macroaggregated albumin (MAA) administration confirms targeted segmental distribution (arrow). (**D**) Digital subtraction angiography during Y-90 microsphere delivery shows an area of staining (arrow). (**E**) Contrast-enhanced CT at 2-month follow-up shows partial necrosis and decreased enhancement within the treated tumor (arrow). (**F**) Magnetic resonance imaging (MRI) at 26-month follow-up demonstrates marked tumor shrinkage (arrow) and treatment response.

TARE with Y-90 microspheres [Figure 6] is one of the most frequently studied IR modalities for unresectable iCCA. Because TARE produces tumor necrosis primarily through internal radiation rather than arterial occlusion, it may be useful for liver-dominant iCCA despite the relative hypovascularity and desmoplastic stroma that can limit embolization-based therapies. Appropriate candidates include patients with unresectable, recurrent, or chemotherapy-refractory disease with preserved hepatic function.

A systematic review and meta-analysis of 21 studies, including 921 patients, reported a disease-control rate of 82.3%, a median progression-free survival of 7.8 months, and a median overall survival of 12.7 months after TARE [67]. A multicenter retrospective study of 81 patients similarly reported a disease-control rate of 83.6% and median overall survival of 14.5 months, with worse survival among patients with tumor burden >50%, neutrophil-to-lymphocyte ratio ≥3, or radiologic progression as the best response [68].

TARE may also have a role in downstaging selected patients to resection, although this is achieved in a minority of cases and depends on tumor distribution, contralateral liver reserve, vascular anatomy, and multidisciplinary surgical assessment. Approximately 10–15% of patients may be successfully downstaged to surgery, which offers the possibility of long-term survival [67]. Toxicity is generally acceptable and includes fatigue, abdominal pain, transient liver enzyme elevation, biliary injury, hepatic decompensation, and radioembolization-induced liver disease, particularly in patients with advanced liver dysfunction or extensive treated liver volume. Comparative studies show similar survival with TARE and TACE in selected unresectable iCCA cohorts [65].

### 3.4. Other Modalities

#### 3.4.1. External Beam Radiotherapy (EBRT) and Stereotactic Body Radiotherapy (SBRT)

EBRT and SBRT are increasingly used in unresectable or recurrent iCCA, including cases with positive margins after resection. Reported median overall survival ranges from approximately 7 to 39 months across heterogeneous series, with local control rates exceeding 80% when high biologically effective doses (>80 Gy) are delivered. For example, Smart et al. [69] reported a 2-year survival of 58% and local control of 84% using hypofractionated EBRT, while Tao et al. [70] demonstrated a 3-year survival of 73% when dose escalation was achieved. Toxicity is generally acceptable but may include fatigue, gastrointestinal injury, hepatic dysfunction, and radiation-induced liver disease, particularly in patients with limited hepatic reserve.

#### 3.4.2. Hepatic Arterial Infusion (HAI)

HAI therapy delivers high concentrations of chemotherapy directly to liver-dominant disease while limiting systemic exposure. In a phase II study of HAI floxuridine combined with systemic gemcitabine and oxaliplatin, the partial response rate was 58%, the 6-month disease-control rate was 84%, median progression-free survival was 11.8 months, and median overall survival was 25.0 months [71]. Catheter-related complications, including infection, thrombosis, and pump malfunction, limit widespread use, but HAI remains an option at specialized centers.

#### 3.4.3. Irreversible Electroporation (IRE) and High-Dose Rate (HDR) Brachytherapy

IRE is particularly suited for centrally located tumors adjacent to major bile ducts or vascular structures where thermal ablation carries a higher risk. However, iCCA-specific evidence remains limited, and robust local-control or survival estimates have not been established. HDR brachytherapy, using CT-guided iridium-192 catheters, is another specialized option for unresectable or recurrent iCCA, particularly for lesions unsuitable for thermal ablation because of size or location. Small retrospective series suggest that durable local control may be achieved, but the evidence remains limited and highly selected [72].

#### 3.4.4. Endobiliary Therapies

Endobiliary therapies, particularly intraductal radiofrequency ablation combined with biliary stenting, are increasingly recognized for palliation in malignant biliary obstruction. Percutaneous endobiliary RFA using the Habib EndoHPB probe has been reported to be technically feasible and safe in unresectable cholangiocarcinoma, with favorable short-term laboratory and clinical outcomes [73]. Meta-analyses suggest that intraductal RFA may prolong stent patency compared with stenting alone, without a clear increase in overall adverse events, although survival findings remain inconsistent [74]. These therapies may improve biliary drainage, reduce recurrent obstruction, and help preserve eligibility for systemic therapy, but they should be considered complementary to systemic and liver-directed tumor treatment.

Representative studies evaluating locoregional and combined-modality therapies for iCCA are summarized in Table 1. The available evidence consists predominantly of retrospective cohorts, single-arm prospective trials, and meta-analyses of heterogeneous observational studies, with limited randomized comparative data. Outcomes should not be directly compared across studies because of substantial differences in disease stage, tumor burden, prior treatment, liver function, treatment technique, response criteria, and patient selection.

## 4. Combined Hepatocellular Cholangiocarcinoma (cHCC–CCA)

Combined hepatocellular cholangiocarcinoma (cHCC-CCA) is a rare primary liver malignancy characterized by unequivocal hepatocytic and cholangiocytic differentiation within the same tumor. Imaging may demonstrate a mixed phenotype, with arterial-phase hyperenhancement resembling HCC and progressive or delayed enhancement reflecting fibrotic cholangiocarcinoma-like components; however, definitive diagnosis generally requires histopathologic confirmation [76]. cHCC-CCA is associated with aggressive behavior and a high risk of recurrence. Outcomes are generally worse than those of HCC, although comparisons with iCCA are inconsistent. Molecular studies demonstrate heterogeneous genomic and transcriptomic profiles, including progenitor-cell and stemness-associated signatures, supporting cHCC-CCA as a biologically distinct entity rather than a simple coexistence of HCC and iCCA [76].

Management of cHCC-CCA is challenging given the absence of standardized guidelines. Surgical resection is the preferred curative-intent treatment for localized disease but is rarely feasible due to late presentation, and recurrence is common. Locoregional therapies may be considered for unresectable, recurrent, or liver-dominant disease, but the evidence remains limited. TACE and TARE exhibit variable efficacy depending on the dominant histologic component. HCC-like hypervascular regions often respond better to embolization [77], whereas iCCA-like fibrotic regions may respond more favorably to radiation-based therapies such as TARE or brachytherapy. TARE has shown encouraging activity in small retrospective cohorts of unresectable cHCC-CCA [78,79]. Ablation may be considered for small tumors, but recurrence rates may be high due to microscopic spread and satellite nodules. Treatment should therefore be individualized through multidisciplinary assessment, incorporating tumor distribution, vascularity, hepatic reserve, extrahepatic disease, and the relative hepatocellular and cholangiocytic phenotype.

## 5. Interventional Radiology in Preoperative Liver Optimization

Major hepatic resection may be precluded not only by tumor extent but also by an inadequate future liver remnant (FLR), impaired regenerative capacity, or clinically significant portal hypertension. In this setting, interventional radiology can expand surgical candidacy through procedures that augment FLR volume and function or optimize portal hemodynamics before resection.

Portal vein embolization (PVE) is an established preoperative strategy for patients in whom the anticipated FLR is considered insufficient for safe major hepatectomy. Selective embolization of the portal venous branches supplying the liver segments planned for resection redirects portal inflow toward the FLR, resulting in atrophy of the embolized liver and compensatory hypertrophy of the nonembolized liver. The minimum acceptable FLR depends on the quality and function of the underlying hepatic parenchyma, with more stringent thresholds in patients with cirrhosis, chronic cholestasis, or treatment-related hepatic injury. Following PVE, cross-sectional volumetry and, when available, functional assessment are used to determine whether sufficient hypertrophy has occurred to permit resection [80]. Liver venous deprivation (LVD), also described as double-vein embolization, combines embolization of the portal vein with embolization of the ipsilateral hepatic vein. By simultaneously interrupting portal inflow and hepatic venous outflow from the portion of the liver planned for resection, LVD may produce more rapid and pronounced FLR hypertrophy than PVE alone. Comparative studies have reported greater hypertrophy and faster kinetic growth after LVD, with fewer patients unable to proceed to surgery because of an inadequate regenerative response. However, available data remain predominantly retrospective, non-randomized, and procedural techniques, and patient-selection criteria have not yet been fully standardized [81,82].

Lobar transarterial radioembolization, commonly termed radiation lobectomy, provides a distinct approach to preparing patients for potential hepatic resection. Unlike PVE or LVD, radiation lobectomy simultaneously treats the tumor-bearing lobe while inducing progressive atrophy of the treated liver and compensatory hypertrophy of the contralateral FLR. Hypertrophy generally develops more slowly than after PVE, but the interval before surgery provides continued local tumor control and a biologic ‘test of time’, during which patients with rapidly progressive intrahepatic or extrahepatic disease can be identified before undergoing major surgery. This approach may therefore be particularly useful in select patients with HCC or iCCA in whom both tumor control and FLR augmentation are required [83,84].

Preoperative transjugular intrahepatic portosystemic shunt (TIPS) placement has also been proposed for carefully selected patients with cirrhosis and clinically significant portal hypertension who would otherwise face prohibitive operative risk. Portal decompression may reduce postoperative ascites and hepatic decompensation, and observational data suggest that preoperative TIPS may lower the risk of postoperative acute-on-chronic liver failure in selected high-risk patients. Portosystemic shunt creation also appears technically feasible and clinically effective in patients with hepatic malignancy; in a recent retrospective series of 39 patients undergoing TIPS or DIPS, technical and clinical success rates were 100% and 92.3%, respectively [85]. However, this study was not limited to preoperative patients and therefore supports feasibility in hepatic malignancy rather than preoperative benefit specifically. Preoperative TIPS should not be considered routine and must be balanced against the risks of hepatic encephalopathy, deterioration in liver function, and altered portal perfusion [86,87].

Selection among PVE, LVD, radiation lobectomy, and preoperative portal decompression should incorporate tumor biology and distribution, the planned extent of resection, FLR volume and function, the severity of underlying liver disease and portal hypertension, and the anticipated timing of surgery and systemic therapy.

Beyond these technical and surgical applications, locoregional therapies may also produce systemic biological effects through modulation of the tumor immune microenvironment, as discussed in the following section.

## 6. Immunologic Effects of Locoregional Therapies and Integration with Systemic Therapy

Locoregional therapies can modify the tumor immune microenvironment in addition to producing direct cytoreduction. Treatment-related cellular injury may release tumor-associated antigens and damage-associated molecular patterns, activate antigen-presenting cells, and stimulate local or systemic tumor-specific immune responses. However, these effects are not uniformly antitumoral. Incomplete tumor destruction, ischemia, and treatment-related inflammation may also induce hypoxia, angiogenesis, compensatory checkpoint signaling, and recruitment of immunosuppressive cell populations. The resulting balance depends on the treatment modality, completeness of tumor destruction, underlying liver disease, tumor biology, and timing relative to systemic therapy [10].

In HCC, RFA has been shown to increase antigen-presenting-cell infiltration and amplify tumor-specific immunity in preclinical models [88]. In a clinical study, RFA enhanced multiple tumor-associated-antigen-specific T-cell responses, and stronger post-treatment responses were associated with longer recurrence-free survival [89]. RFA has also been associated with activation of natural killer cells [90]. Conversely, residual viable tumor exposed to sublethal heat may activate HIF-1α/VEGF-mediated and inflammatory pathways, supporting the importance of complete ablation and adequate margins [10].

MWA produces similar, although variable, immune effects. Sequential tissue analysis after MWA demonstrated increased lymphocytic and natural-killer-cell infiltration within treated and distant untreated HCC lesions [91]. MWA has also been associated with enhanced tumor-specific immune responses in patients with HCC [92]. A phase I study combining MWA with adoptive cellular immunotherapy reported a reduction in regulatory T cells and an increase in circulating effector T-cell populations, but this study did not evaluate checkpoint inhibition [93]. Separate clinical data demonstrated increased IL-12 and reduced IL-4 and IL-10 after MWA, consistent with a shift toward a Th1-associated profile [94]. These findings support combination strategies, although prospective evidence with immune checkpoint inhibitors remains limited.

Cryoablation may preserve structurally intact antigenic material and can generate both stimulatory and suppressive immune responses. In HBV-associated HCC, higher circulating PD-1 and PD-L1 expression, which correlated with intratumoral PD-L1 expression, was associated with poorer outcomes after cryoablation [95]. Clinical studies combining cryoablation with adoptive natural-killer-cell or dendritic-cell/cytokine-induced-killer-cell therapy have suggested improved outcomes, but these were small, nonrandomized studies and do not establish synergy with contemporary checkpoint inhibitors [96,97].

Nonthermal approaches may have distinct immunologic effects. In patients with HCC, IRE increased activated circulating T cells and natural-killer cells while reducing regulatory T cells; complementary animal experiments demonstrated increased cytotoxic CD8-positive T-cell infiltration and IFN-γ production [98]. Histotripsy has produced systemic immune activation and increased distant-tumor CD8-positive T-cell infiltration in preclinical models, but clinical immunologic evidence in primary liver malignancies remains limited [50].

TACE also produces competing immune effects. A prospective clinical study demonstrated reductions in regulatory T-cell populations after TACE, and high post-treatment regulatory T-cell levels were associated with shorter time to progression [99]. Changes in circulating cytokines and immunogenic-cell-death biomarkers, including HMGB1, have also been reported after treatment [100,101]. Conversely, embolization-induced hypoxia may increase HIF-1α and VEGF signaling in surviving tumors, providing a biologic rationale for combinations with antiangiogenic agents and immune checkpoint inhibitors [10]. The optimal sequence and patient population for these combinations remain under evaluation.

Y-90 radioembolization has similarly demonstrated local and systemic immune modulation. Immune profiling of HCC after radioembolization showed increased intratumoral granzyme B expression and infiltration by CD8-positive T cells, CD56-positive natural-killer cells, and natural-killer T cells, together with systemic activation of antigen-presenting cells and T-cell TNF-α expression [102]. Radioembolization may also produce transient lymphopenia and impaired lymphocyte function [103]. In parallel, increases in IL-6, IL-8, von Willebrand factor, plasminogen activator inhibitor-1, factor VIII, and D-dimer indicate systemic inflammation, endothelial stress, and coagulation activation [104]. These findings provide a rationale for integration with checkpoint inhibition; a phase II study of Y-90 radioembolization followed by nivolumab reported an objective response rate of 30.6%, although the study was single-arm and did not establish superiority over either treatment alone [105].

The evidence linking locoregional treatment to clinically meaningful immune activation is strongest in HCC. In iCCA, dense desmoplastic stroma and an immune-excluded microenvironment may limit immune-cell penetration, and direct evidence that locoregional treatment enhances immunotherapy responsiveness remains sparse. cHCC–CCA is also immunologically heterogeneous. Primary immune profiling identified immune-high and immune-low subtypes, with the immune-high subtype demonstrating greater immune-cell density and enrichment of immune-response signatures [106]. However, these findings do not yet establish that locoregional therapy improves checkpoint-inhibitor efficacy in either iCCA or cHCC–CCA.

Overall, locoregional therapy may function as both a cytoreductive and immune-modulating intervention, but the magnitude and clinical relevance of these effects vary by modality and tumor type. Prospective studies integrating tissue sampling, circulating biomarkers, treatment completeness, dosimetry, and systemic-therapy sequencing are needed to identify patients most likely to benefit from combined locoregional and immunotherapeutic strategies.

## 7. Conclusions

Interventional radiology is integral to the multidisciplinary management of primary liver malignancies, providing curative-intent, downstaging, bridging, palliative, and preoperative treatment options across HCC, iCCA, and cHCC–CCA. Ablation and transarterial therapies are well established in HCC, while in iCCA they can provide meaningful disease control and complement systemic therapy in carefully selected patients. In cHCC–CCA, treatment remains individualized because of heterogeneous tumor biology and limited prospective evidence. Interventional radiology also expands surgical candidacy through portal vein embolization, liver venous deprivation, radiation lobectomy, and, in selected patients, portal decompression before resection.

Future advances will depend on more precise patient selection, optimized treatment techniques and dosimetry, and closer integration of locoregional therapy with systemic and immune-based treatments. Prospective comparative studies incorporating imaging findings, dosimetric parameters, and molecular and immunologic biomarkers are needed to define treatment sequencing, identify patients most likely to benefit, and improve oncologic outcomes while preserving hepatic function.

## Figures and Tables

**Table 1:** Representative Studies of Locoregional and Combined-Modality Therapies for Intrahepatic Cholangiocarcinoma (iCCA).

Modality	Study	Study Design and Population	Intervention	Key Findings	Evidence Level and Principal Limitations
Thermal ablation	Kim et al., 2022 [58]	Systematic review and meta-analysis of 20 observational studies; 917 patients with primary or recurrent iCCA	RFA or MWA	Pooled technical efficacy was 91.9%. Pooled 1-, 3-, and 5-year OS rates were 82.4%, 42.1%, and 28.5%, respectively; the major complication rate was 5.7%. Tumors >3 cm and multifocal disease were associated with worse survival.	Meta-analysis of observational studies; heterogeneous techniques, tumor burden, and primary vs. recurrent disease.
Thermal ablation	Briot et al., 2024 [63]	Retrospective multicenter cohort; 24 patients with one or two iCCA lesions ≤35 mm	Monopolar RFA or MWA	Median disease-free survival was 10.5 months, and median OS was 40.8 months.	Small, highly selected retrospective cohort; mixed RFA and MWA; no untreated or surgical control group.
Thermal ablation vs. repeat surgery	Xu et al., 2019 [62]	Retrospective comparative study of recurrent iCCA after hepatectomy	Percutaneous MWA vs. repeat surgical resection	Estimated 5-year OS was 23.7% after MWA and 21.8% after repeat resection. Major complications occurred in 5.3% and 13.8%, respectively.	Nonrandomized comparison; selection bias and limited generalizability to primary or extensively recurrent disease.
TACE	He et al., 2023 [64]	Systematic review and pooled analysis of 19 studies comprising 23 cohorts and 1091 patients with unresectable iCCA	Conventional TACE or DEB-TACE	Pooled objective response rates were 29.4% with cTACE and 51.2% with DEB-TACE; disease-control rates were 72.8% and 88.7%, respectively. A significant overall survival advantage with DEB-TACE was not demonstrated.	Pooled observational evidence; predominantly indirect comparison between heterogeneous cTACE and DEB-TACE cohorts.
TACE vs. TARE	Mosconi et al., 2021 [65]	Systematic review and meta-analysis of 31 studies including 1695 patients with unresectable iCCA; 13 TACE studies included 906 patients, and 18 TARE studies included 789 patients	TACE or Y-90 TARE	Median overall survival was 14.2 months after TACE and 13.5 months after TARE. Clinical adverse events were less frequent after TARE than TACE, at 43.0% vs. 58.5%, respectively.	Indirect comparison of predominantly retrospective studies; heterogeneity in patient selection, technique, and outcome assessment.
TARE	Schartz et al., 2022 [67]	Systematic review and meta-analysis of 21 studies; 921 patients with unresectable iCCA	Y-90 TARE	Disease-control rate was 82.3%; median PFS was 7.8 months, and median OS was 12.7 months. Approximately 11.0% of patients were downstaged to surgical resection.	Meta-analysis of mainly retrospective, single-arm studies; variable microsphere platforms and dosimetry.
TARE plus systemic chemotherapy	Edeline et al., 2020; MISPHEC [75]	Prospective multicenter phase II trial; 41 treatment-naïve patients with unresectable locally advanced iCCA	Y-90 TARE plus first-line gemcitabine–cisplatin	RECIST response rate was 39% at 3 months, and best central response was 41%; and disease-control rate was 98%. Median PFS and OS were 14 and 22 months, respectively. Nine patients (22%) were downstaged to surgery, including eight who achieved R0 resection.	Prospective phase II evidence, but single-arm and small; Grade 3–4 toxic effects occurred in 71% of patients.
Dose-escalated EBRT	Tao et al., 2016 [70]	Single-center retrospective dose–response analysis of 79 patients with inoperable iCCA	Dose-escalated external-beam radiotherapy	A biologically effective dose >80.5 Gy was associated with a 3-year OS of 73%, compared with 38% after lower-dose treatment; improved local control was also observed with dose escalation.	Retrospective single-center study; treatment-selection and dose-selection bias; heterogeneous systemic therapy.
Hypofractionated EBRT	Smart et al., 2020 [69]	Retrospective cohort of 66 patients with unresectable or locally recurrent iCCA	Hypofractionated photon or proton radiotherapy	Reported 2-year local control and OS were 84% and 58%, respectively; among patients treated with definitive intent, corresponding rates were 93% and 62%.	Retrospective cohort; heterogeneity in radiation technique, dose, and treatment intent.
Hepatic arterial infusion plus systemic chemotherapy	Cercek et al., 2020 [71]	Prospective phase II trial of 38 patients with unresectable iCCA	HAI floxuridine plus systemic gemcitabine and oxaliplatin	Partial response rate was 58%; 6-month disease-control rate was 84%. Median PFS and OS were 11.8 and 25.0 months, respectively, and 1-year OS was 89.5%. Four patients underwent subsequent resection.	Prospective phase II evidence, but single-arm, specialized-center treatment, and limited external generalizability.

Abbreviations: cTACE, conventional transarterial chemoembolization; DEB-TACE, drug-eluting bead transarterial chemoembolization; EBRT, external-beam radiotherapy; HAI, hepatic arterial infusion; iCCA, intrahepatic cholangiocarcinoma; MWA, microwave ablation; OS, overall survival; PFS, progression-free survival; RECIST, Response Evaluation Criteria in Solid Tumors; RFA, radiofrequency ablation; TACE, transarterial chemoembolization; TARE, transarterial radioembolization.

## Data Availability

No new data were created or analyzed in this study. Data sharing does not apply to this article.
